# Structural Characterization and Electrochemical Performance of Apricot Kernel-Based Activated Carbons for Supercapacitors

**DOI:** 10.1021/acsomega.5c00919

**Published:** 2025-07-29

**Authors:** Elif Onyilmaz, Yunus Onal, Erdinc Oz

**Affiliations:** 1 Department of Energy Science and Technologies, 37520 İnönü University, Malatya 44280, Türkiye; 2 Department of Chemistry, İnönü University, Malatya 44280, Türkiye; 3 Department of Physics, 37503 Atatürk University, Erzurum 25240, Türkiye; 4 Department of Nanoscience and Nanoengineering, Atatürk University, Erzurum 25240, Türkiye

## Abstract

Rapid industrialization and population growth have intensified global energy demands, emphasizing the need for sustainable and efficient energy storage systems. This study investigates the utilization of oil-extracted apricot kernels as a biomass precursor for activated carbon synthesis. Through carbonization and activation, two samples (ASE3 and ASE5-T) were produced and characterized using XRD, FTIR, Raman spectroscopy, SEM/EDX, BET analysis, and electrochemical testing. Among the samples, ASE3 demonstrated superior structural and electrochemical properties, highlighting its potential for energy storage applications. Structural studies revealed a micropore-dominated structure with hierarchical porosity, optimizing ion transport and charge storage. This approach provides a sustainable solution to waste management and paves the way for cost-effective alternatives to traditional energy storage materials. Future studies should explore the long-term cycling stability, the potential for industrial-scale production, and hybrid system integrations for energy storage solutions.

## Graphical abstract

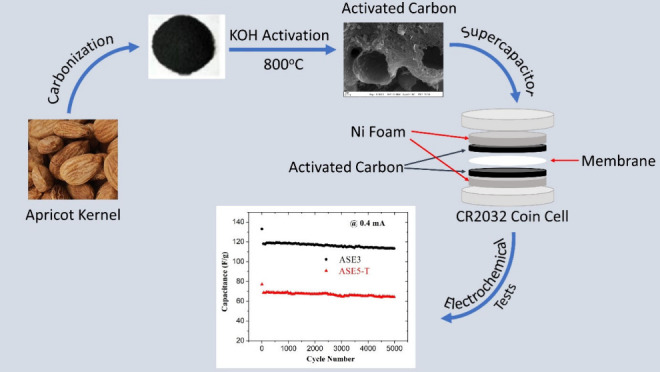

## 1 Introduction

The accelerating pace of industrialization and population growth has substantially increased global energy consumption, creating a pressing imbalance between energy production and demand. This imbalance necessitates the efficient utilization of existing energy resources. Fossil fuels have traditionally served as the primary energy source; however, their detrimental environmental impact and finite reserves have intensified the global pursuit of sustainable energy alternatives. Renewable energy sources provide a cleaner and more sustainable alternative, yet their variable and intermittent nature, influenced by environmental factors such as weather, highlights the critical need for robust energy storage systems. Ensuring a stable and continuous energy supply requires technologies that can store excess energy when production surpasses demand and release it during shortages. Unlike fossil fuels, renewable energy sources offer environmentally benign solutions that do not compromise ecological integrity.^1^

Renewable energy systems inherently face fluctuations due to environmental variability, necessitating advanced energy storage solutions to stabilize supply and meet demand during peak periods. Diverse energy storage methods have been developed to address these challenges, including batteries, flow batteries, thermal energy storage, and electrochemical capacitors. While batteries are well-suited for long-term energy storage, they often fail to deliver the instantaneous power required for certain applications. Consequently, electrochemical capacitors, such as electric double-layer capacitors (EDLCs) and pseudocapacitors, serve as critical components in renewable energy systems. EDLCs store charge through the physical adsorption of electrolyte ions on the electrode surface without involving redox reactions, allowing for rapid and efficient responses to potential changes.^2–6^ This unique capability makes them indispensable for managing sudden energy surges.

Among various electrochemical energy storage devices, supercapacitors have gained attention due to their high-power density, long cycle life, and environmental compatibility. These devices rely heavily on the choice of electrode materials, which significantly influence their performance.^7–9^ Activated carbon-based supercapacitors have emerged as a leading class due to their exceptional structural properties and widespread availability.^10^ To further enhance their applicability, there is a pressing need to develop low-cost, sustainable, and widely available electrode materials.^11^ Carbon-based electrodes, particularly those derived from biomass, offer numerous advantages, including high surface area, excellent electrical conductivity, ease of fabrication, low production cost, and robust chemical stability.^12,13^ Activated carbon (AC), with its expansive internal surface area and optimized micropore- macropore architecture, stands out as a highly versatile adsorbent.^14,15^ Its exceptional adsorptive properties not only facilitate efficient ion transport in energy storage systems but also render it suitable for diverse applications, such as pollutant removal, toxin filtration, water purification, salt removal, and catalytic processes.

Activated carbon is widely valued for its high porosity, large surface area, and superior adsorption capacity, making it a crucial material for energy storage applications.^16,17^ Biomass has emerged as an ideal precursor for activated carbon due to its abundance, renewability, and cost-effectiveness.^18^ Unlike conventional carbon sources, such as coal and petroleum-based materials, biomass-based activated carbon offers significant environmental advantages, including reduced greenhouse gas emissions and enhanced sustainability.^19^ Various biomass sources, including agricultural waste, fruit shells, and nutshells, have been successfully utilized for activated carbon production, demonstrating remarkable electrochemical properties in supercapacitor applications.^20^

Among various biomass options, apricot kernels stand out due to their high carbon content and favorable structural properties. Apricot Kernel-Based Activated Carbons (AKACs) offer several advantages over previously studied biomass-derived carbons. First, apricot kernels, as an agricultural waste product, provide a cost-effective and sustainable precursor for activated carbon synthesis.^21^ Unlike coconut shells, bamboo stalks, or walnut shells, which require additional processing steps, apricot kernels contain a high fixed carbon content and a favorable micropore-to-mesopore ratio, enhancing ion transport and charge storage capacity. Studies have shown that coconut shell-derived activated carbon exhibits a specific capacitance of approximately 175.6 F/g, while bamboo-based activated carbon reaches 222 F/g, and garlic skin-derived carbon demonstrates an impressive 315 F/g.^22^ However, AKACs, particularly those synthesized through chemical activation methods, have demonstrated comparable or superior electrochemical performance, achieving specific capacitance values of up to 339 F/g in some studies.^23^ Additionally, the oil extraction process in apricot kernels disrupts their cellular structure, leading to improved chemical activation efficiency and the formation of hierarchical porous networks, further enhancing ion diffusion and storage capacity. These features make AKACs a strong candidate for high-performance supercapacitor applications. In this context, apricot kernels, abundantly sourced from the Malatya region, were utilized as the primary raw material for AC production. Following oil extraction, these kernels exhibit a disrupted cellular structure rich in diverse organic functional groups, making them highly reactive and suitable for chemical modifications. The resulting activated carbon structures possess desirable characteristics, including high carbon content, low ash content, and superior mechanical strength, which collectively enhance ion transfer efficiency and energy storage capacity.

In contrast to previous studies that primarily utilize apricot shells, this work introduces defatted apricot kernels, an underexplored and compositionally distinct biomass precursor, as the carbon source. Defatted kernels provide a denser carbon matrix with a unique composition (protein- and carbohydrate-rich), enabling distinct pore development and structural features. Additionally, the synthesis route combines precarbonization with CO_2_ activation, offering an energy-efficient and sustainable approach with promising electrochemical performance.

The findings revealed that these materials exhibit excellent potential as supercapacitor electrodes, providing a sustainable solution for advanced energy storage systems.

## 2 Results and Discussion

### 2.1 Structural Properties

Figure 1 shows the XRD patterns of ASE3 and ASE5-T samples. Both samples exhibit broad P1 and P2 peaks in the 11–35° 2θ range, characteristic of amorphous materials lacking long-range crystalline order. These findings suggest the presence of short-range ordered, low-graphitic carbon structures, consistent with previous reports.^24–26^

**Figure 1:**
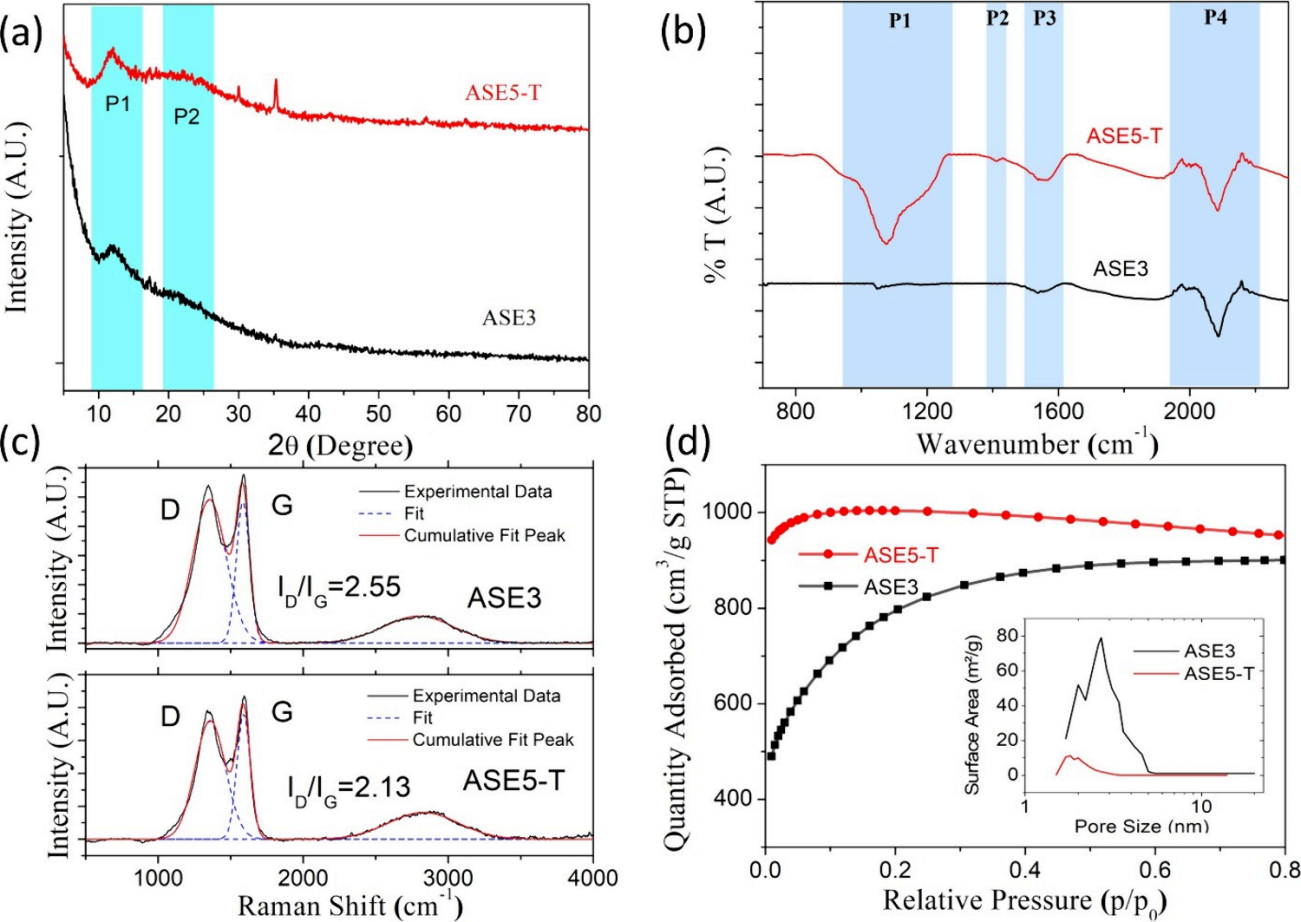
(a) XRD pattern of ASE3 (black line) and ASE5-T (red line). (b) FTIR spectra of ASE5-T (red) and ASE3 (black) samples in the mid-infrared region. Four distinct spectral regions (P1–P4) are highlighted, indicating similarities in functional groups between the two samples. (c) Raman spectra of ASE3 and ASE5-T samples fitted with Gaussian deconvolution. The D band (∼1350 cm^–1^) corresponds to disordered carbon structures, while the G band (∼1580 cm^–1^) indicates graphitic sp^2^ domains. The calculated *I*_D_/*I*_G_ ratios are 2.55 for ASE3 and 2.13 for ASE5-T, suggesting a higher degree of structural disorder in ASE3. (d) Nitrogen adsorption–desorption isotherms of ASE3 and ASE5-T samples. The inset figure is the pore size distribution curves of ASE3 and ASE5-T samples.

Given that the synthesis process in this study involves carbonization at 400–500 °C and chemical activation at 800 °C without oxidative exfoliation treatments, the formation of graphene oxide under these conditions is unlikely. Therefore, the peak at 43.0° is more appropriately attributed to the (002) plane of low-crystallinity graphitic carbon domains, consistent with the structural evolution described in biomass-derived graphitic carbons subjected to high-temperature treatments,^27^ often observed in biomass-derived carbons subjected to KOH activation. These broad, low-intensity peaks reflect the presence of turbostratic or disordered stacked carbon layers rather than well-defined graphene or GO structures.^28,29^

Additionally, ASE3 exhibits sharper peaks superimposed on the amorphous background, which may indicate partial graphitization induced during activation.^30,31^ In contrast, ASE5-T shows some crystalline reflections at ∼28° and ∼35°, likely originating from residual inorganic impurities due to incomplete carbonization.^32^ These results suggest that both samples contain graphitic domains to varying extents, shaped by their respective synthesis conditions.

Figure 1b presents the Fourier Transform Infrared (FTIR) spectra of samples prepared with varying compositions. FTIR spectroscopy enables the identification of functional groups within a sample by analyzing the absorption of infrared radiation. The absorbance values for each sample are summarized in Table 1. FTIR analysis was conducted in the 400–4000 cm^–1^ range, with key functional group peaks observed in the mid-infrared region.

**Table 1:** Wavelengths of the Peaks Observed in the FTIR Spectrum of ASE3 and ASE5-T samples

sample	P1	P2	P3	P4
ASE3	1046	1405	1566	2090
ASE5-T	1074	1412	1550	2082

Four distinct spectral regions (P1–P4) were identified in Figure 1(b). The P1 peak (950–1300 cm^–1^) is attributed to C–O stretching vibrations, likely from alkanes, alcohols, and phenols in the raw material. The P2 peak (1000–1450 cm^–1^) is associated with C–C stretching vibrations. The P3 peak (1500–1650 cm^–1^) is assigned to C=O and C=C stretching vibrations, indicative of carbonyl and olefinic groups, respectively. Finally, the P4 peak (1900–2400 cm^–1^) is related to C=O and C–O stretching vibrations, which are characteristic of aromatic ring structures within the lignin component.^33–35^ Notably, the FTIR spectra of activated carbons carbonized at elevated temperatures exhibit simpler profiles. This simplification may be attributed to increased carbonization, which results in a more amorphous carbon structure.

Figure 1c presents the Raman spectra of the samples, revealing three distinct bands: the D-band (1200–1400 cm^–1^), the G-band (1400–1800 cm^–1^), and the 2D-band (2700–2850 cm^–1^). These bands are characteristic of carbon-based materials. The relative intensities of these bands provide insights into the degree of graphitization and the presence of defects within the carbon structure.^36–38^ The “M-shaped” Raman spectrum with the G-band and D-band at 1580 cm^–1^ and 1330 cm^–1^ indicates a carbon structure with balanced graphitic and disordered carbon components.^39^ The Raman spectrum of ASE3 exhibits distinct D, G, and 2D bands in the expected regions. The broadening and decrease in intensity of the 2D band, with the appearance of an M-shaped profile in the D and G bands, indicate the presence of both graphitic and disordered carbon structures within the ASE3 sample. The D-band, a consequence of structural defects and disorder, can be associated with C–C bonds.

Raman spectra provide insights into the degree of graphitization and the presence of defects within the carbon structure. The distinct D-band (∼1330 cm^–1^) and G-band (∼1580 cm^–1^), along with a broadened, low-intensity 2D band (∼2700–2850 cm^–1^) observed in the ASE3 sample, suggest the coexistence of disordered sp^3^-hybridized carbon and graphitic sp^2^ domains. The broadness of the D and G bands and the absence of a sharp 2D peak, typically present in crystalline graphene, indicate predominantly disordered and defect-rich carbon structures rather than well-defined graphene or GO structures.^40^ These findings support the formation of amorphous structures and defects within the material during carbonization and activation.

Furthermore, the *I*_D_/*I*_G_ ratio, derived from the intensity of the D and G bands, was calculated to be 2.55 for ASE3 and 2.13 for ASE5-T (Figure 1c). This ratio is commonly used to assess the degree of structural disorder in carbon-based materials. The higher *I*_D_/*I*_G_ ratio of ASE3 suggests a greater level of defect density and amorphous character compared to ASE5-T. In contrast, the relatively lower ratio in ASE5-T indicates a higher degree of structural ordering and lower defect concentration. These findings align with the morphological observations and support the interpretation that ASE3 exhibits a more disordered carbon framework, which may influence ion adsorption and charge storage behavior in electrochemical applications.

The specific surface area (SSA), micropore/mesopore ratio, and pore size distribution are critical factors that govern the electrochemical performance of porous carbon-based supercapacitor electrodes. Activated carbons with higher surface areas and optimal pore size distributions facilitate efficient ion transport, improve charge storage capabilities, and enhance overall capacitance. The porous structure of activated carbons is inherently nonuniform and varies depending on synthesis conditions and precursor materials.^41^ BET analysis was conducted to define the microporous structural properties of samples prepared as cathode powders (ASE3 and ASE5-T). The BET analysis results of the synthesized activated carbons are shown in Table 2. The tabulated data indicates that the carbonization temperature significantly influences the specific surface area of the activated carbon samples. The higher-temperature carbonized ASE3 exhibits a notably greater specific surface area (2767.45 m^2^/g) than ASE5-T (1096 m^2^/g), as demonstrated in Figure 1d.

**Table 2:** BET Analysis Data of ASE3 and ASE5-T Samples

material	specific surface area (m^2^/g)	total pore volume (cm^3^/g)	micro pore surface area (m^2^/g)	meso pore surface area (m^2^/g)	micro pore volume (cm^3^/g)	meso pore volume (cm^3^/g)	average pore diameter dp (nm)
ASE3	2767.45	1.410	598.45	2169	0.277	1.133	2.0391
ASE5-T	1096	0.494	636.65	261	0.311	0.183	2.1982

ASE3 exhibits a hierarchical pore structure characterized by a substantial mesopore surface area (2169 m^2^/g) complemented by a notable micropore contribution (598.45 m^2^/g), resulting in a micro/mesoporous architecture that facilitates effective ion transport and energy storage. A more quantitative analysis of the BET results shows that micropores account for approximately 21.6% and mesopores 78.4% of the total surface area, indicating a mesopore-dominant structure rather than a strictly balanced distribution. While micropores serve as primary sites for electric double-layer charge storage, mesopores enhance ion transport and ensure efficient electrolyte accessibility, especially at higher scan rates. Therefore, despite the relatively lower microporosity, the well-developed mesoporous network supports rapid ion diffusion and contributes positively to the overall electrochemical performance of ASE3. This synergistic pore configuration underlines the importance of hierarchical porosity in optimizing both energy and power densities in supercapacitor applications.

In contrast, ASE5-T shows a dominant micropore surface area (636.65 m^2^/g) with a relatively smaller mesopore contribution (261 m^2^/g), which may limit ion transport and reduce overall capacitance. A quantitative analysis indicates that micropores constitute approximately 70.9% of the total surface area, while mesopores account for 29.1%. Although the high microporosity enhances charge storage capacity via electric double-layer formation, the limited mesoporosity may hinder rapid ion diffusion, especially under high-rate conditions. This could explain the lower capacitance and rate capability observed for ASE5-T compared to ASE3. It is evident that increasing the carbonization temperature initially increases surface area, followed by a subsequent decrease beyond a certain threshold. An increased surface area enables enhanced contact between the electrolyte and the electrode, facilitating improved ion diffusion kinetics and potentially higher power density in electrochemical applications.^42^

Figure 2a–d presents scanning electron microscopy (SEM) images of the surface morphology of ASE3 and ASE5-T powders. The corresponding elemental composition, determined via energy-dispersive X-ray spectroscopy (EDX), is depicted in Figure 2b,d and summarized in Figure 2g,h. The SEM micrographs reveal that both activated carbon samples have a porous structure, consistent with the findings of the BET analysis. The presence of these porous structures is evidence of successful activation, a finding further supported by the results of the ash analysis. To optimize the surface area and performance of activated carbons, future research should prioritize the optimization of the synthesis conditions to facilitate the development of a more robust, interconnected porous network.

**Figure 2:**
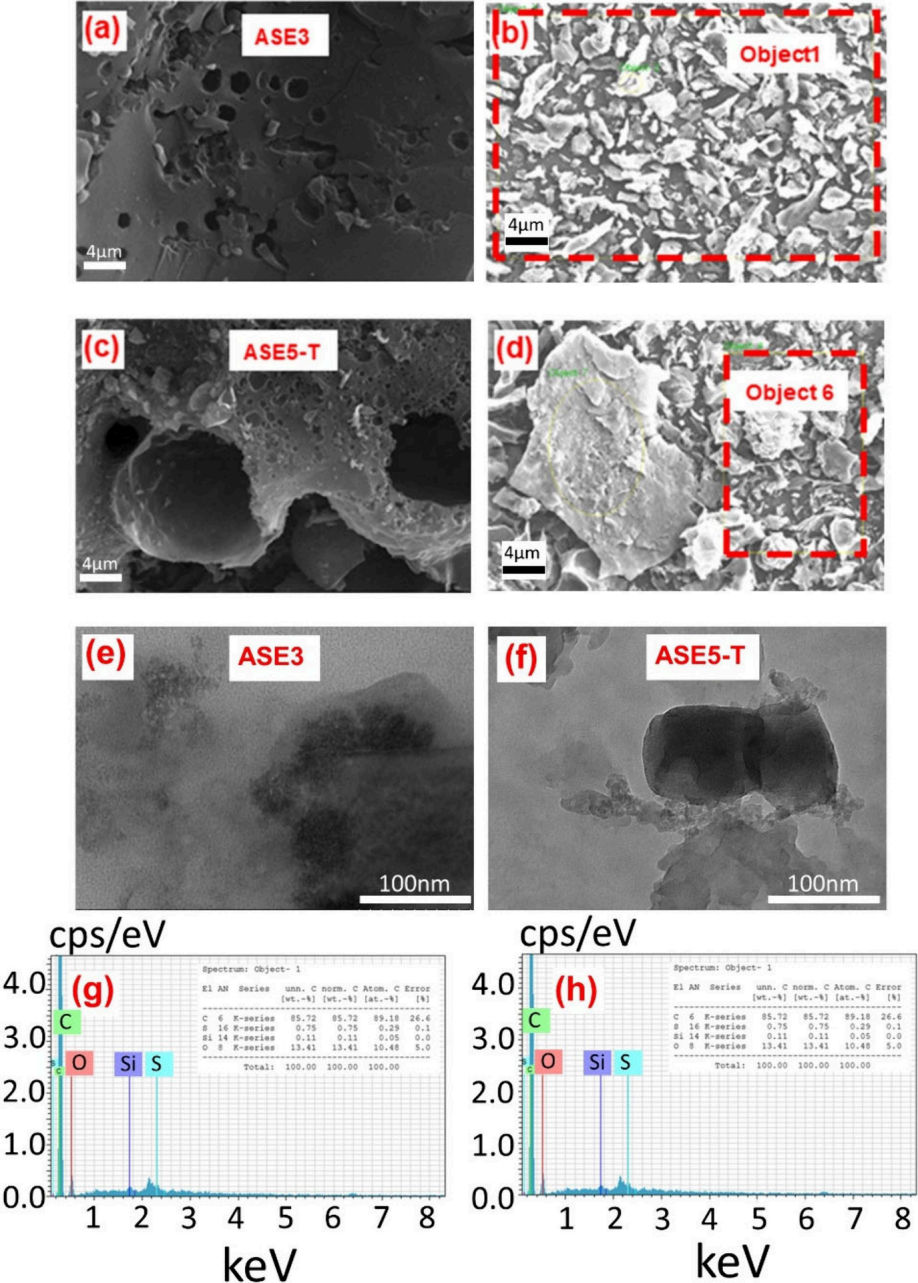
SEM image of ASE3 and ASE5-T samples in (a) and (c), respectively, in 10k magnifications. The areas where EDX analyses of ASE3 and ASE5-T samples were taken are indicated by red dashed lines in (b) and (d), TEM images of (e) ASE3, and (f) ASE5-T. EDX results of the (g) ASE3 and (h) ASE5-T samples.

The TEM images of the ASE3 (Figure 2e) and ASE5-T (Figure 2f) samples reveal distinct differences in morphology, surface area, and transparency, indicative of their structural evolution. ASE3 exhibits a highly transparent and diffuse structure with poorly defined particle boundaries, suggesting a predominantly amorphous and low-density morphology. This high electron transparency implies a large specific surface area, likely advantageous for applications requiring extensive interfacial contact, such as adsorption or ion transport.

In contrast, the ASE5-T sample also displays some transparent, sheet-like regions, although certain areas exhibit a relatively darker contrast. This may be attributed to partial agglomeration or stacking of nanosheets, possibly due to limited sonication during TEM sample preparation. While clearer lattice fringes were initially interpreted as evidence of improved crystallinity, we acknowledge that such fringes are not distinctly visible in Figure 2f. Therefore, rather than making definitive claims about crystallinity, we cautiously suggest that some domains in ASE5-T may reflect partial graphitization and increased particle thickness as a result of thermal treatment. The reduced transparency in these regions corresponds to a relatively denser structure, which may imply a lower surface area but enhanced structural integrity. Elemental analysis via EDX indicates that both ASE3 and ASE5-T are primarily composed of carbon, with respective abundances of 85.72% and 87.70%. These results align well with the observations from SEM and Raman spectroscopy. Table 3 presents the elemental analysis data for the investigated samples. The Si element observed in the EDX plots may originate from trace impurities in the biomass precursor or contamination during sample preparation.

**Table 3:** Elemental Analyses of the Samples

sample	%C (darbon)	%H (hydrogen)	%N (nitrogen)	%S (sulfur)	%O^a^(oxygen)
ASE3	78.29	0.383	0.609		20.718
ASE5-T	66.53	1.283	1.903	0.743	29.541

a Calculated from the difference.

As the structure is adjusted with temperature, groups containing hydrogen, oxygen, nitrogen, and sulfur, other than carbon, move away from the structure. The order and amount of separation depend on the size of the groups in which these molecules are located, whether they are attached to the carbon atom or not, and the size of the macromolecular structure. The high amount of carbon found in the apricot kernel shows that this biomass waste can be used for activated carbon synthesis, and it was found to be quite high for each sample. Trace amounts of sulfur were detected in the elemental analysis of each sample.^43^

Table 4 reveals a high ash content in the raw sample. Interestingly, despite the expected carbon loss during chemical activation, the ash content decreases rather than increases. This phenomenon can be attributed to the transformation of inorganic components within the structure into water-soluble forms, which are subsequently removed during the activation process.

**Table 4:** Ash Determination Analysis Rate of Samples

samples	ash(m/m) (650 °C) %	ash(m/m) (850 °C) %
ASE3	1.93	1.36
ASE5-T	14.56	11.72

A comparative analysis reveals a higher activated carbon yield from high-temperature carbonization processes, while lower yields are observed at lower temperatures. This disparity arises from the extent of structural rearrangement during carbonization. Elevated temperatures induce substantial structural rearrangement, limiting the importance of interaction with added chemical biochar and directing a significant proportion of the material toward forming pores. Conversely, incomplete structural rearrangement at lower temperatures facilitates the reaction of added chemicals with small organic groups within the structure, leading to their consumption. Consequently, lower surface areas and activated carbon yields are obtained. To optimize energy efficiency, minimize the carbon footprint, and align with green initiatives, it is imperative to maintain carbonization temperatures at or above 500 °C and employ chemical activation to achieve substantial activated carbon yields and extensive surface areas.

### 2.2 Electrochemical Properties

Cyclic voltammetry (CV) was employed to investigate the electrochemical performance of the prepared supercapacitors, including their operating voltage windows and redox reactions. The samples were assembled into sandwich-type supercapacitor configurations for CV measurements. The CV curves in Figure 3a.b exhibit a characteristic shape indicative of supercapacitor behavior. The maximum current densities at 1 V for ASE3 and ASE5-T, measured at a scan rate of 200 mV/s, were found to be 0.073 and 0.072 A, respectively. As expected, a decrease in the scan rate from 200 mV/s to 5 mV/s resulted in a corresponding decrease in current density.^44^ The CV curves obtained for the supercapacitors exhibit a quasi-rectangular shape, characteristic of ideal capacitive behavior. The ASE3-based supercapacitor demonstrates superior performance compared to the ASE5-T-based device. As the scan rate increases, the rectangular shape of the CV curves becomes less pronounced. This deviation from ideal behavior is attributed to the limitations in ion diffusion within the porous structure of the activated carbon, particularly at higher scan rates.^45^ The CV curves exhibit a near-ideal rectangular shape at lower scan rates (5–75 mV/s), highlighting efficient ion transport within the materials. However, at higher scan rates (100–200 mV/s), the rectangular shape becomes distorted, suggesting increased resistance to ion diffusion within the carbon matrix. This increased resistance is attributed to the limitations in ion mobility within the porous structure, which is particularly pronounced at higher scan rates. The maintenance of the ideal rectangular shape at lower scan rates suggests rapid and reversible charge storage/discharge processes, consistent with the formation of an electric double layer on the electrode surface. These findings align with previous literature on electrochemical double-layer capacitors.^46^ As the scan rate increases, the current density at the electrode surface also increases. The deviation from ideal capacitive behavior, characterized by a loss of rectangular shape in the CV curves, is expected and becomes more pronounced at higher scan rates and current densities. This behavior is attributed to the hindered ion diffusion kinetics within the porous electrode matrix. Capacitance values for double-layer capacitors were determined using

1 $$C=\int \frac{I\;dV}{2\cdot \Delta v\cdot s\cdot m}$$

where “*C*” is the specific capacitance of the device, “*I*” is the current density, “*t*” is the discharge time, and “*m*” is the mass of active substances, respectively. Capacitance values of ASE3 and ASE5-T samples depending on different scanning speeds were calculated using eq 1, and the resulting graph is given in Figure 3c. The highest specific capacitances were 136 F/g for ASE3 and 75.5 F/g for ASE5-T at a scan rate of 5 mV/s. As expected, the specific capacitance decreases with increasing scan rate, a common trend observed in capacitor systems. A comparison of these values with those reported in the literature for biomass-based capacitors (Table 5) reveals that the synthesized materials exhibit promising performance.

**Figure 3:**
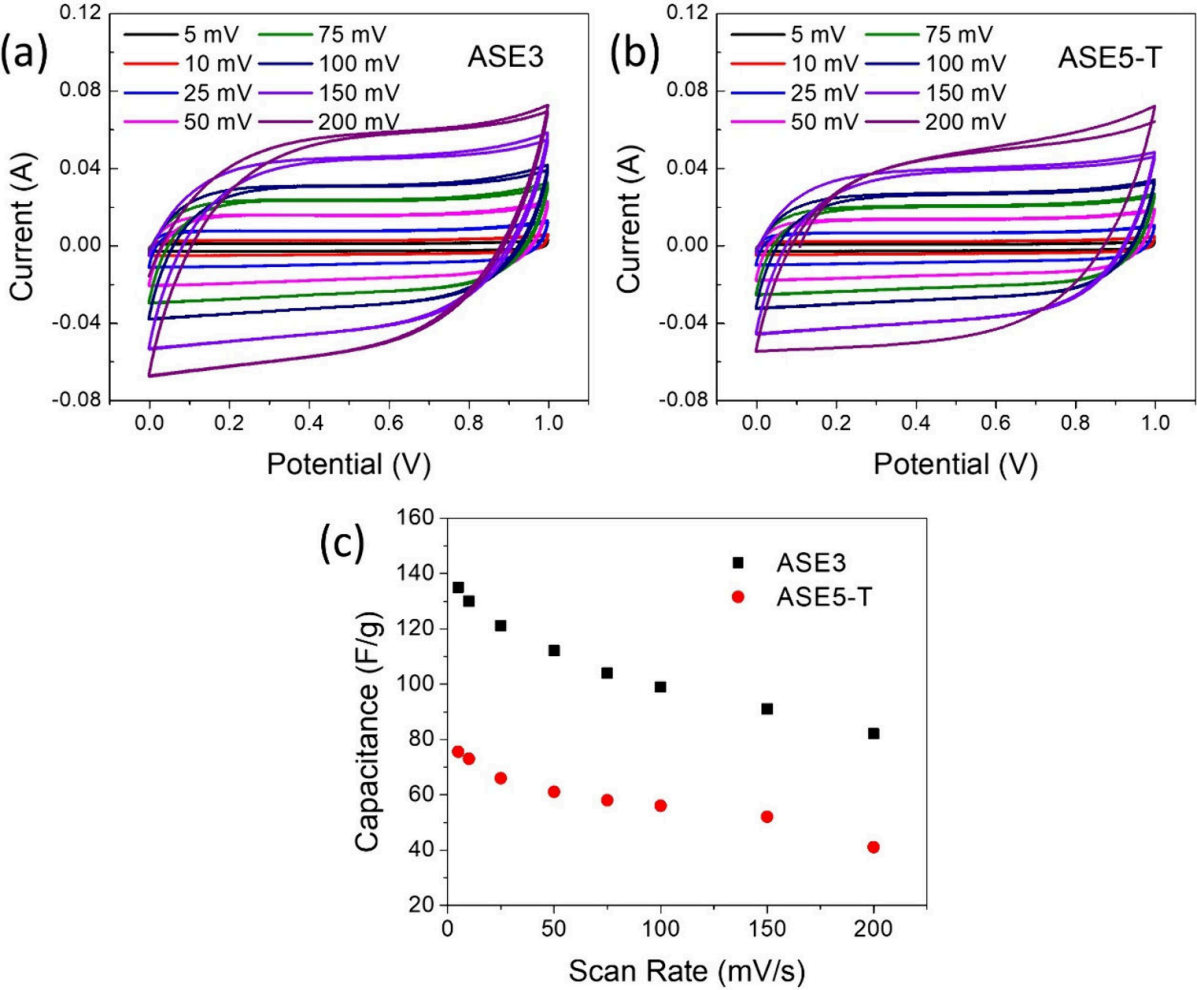
Potential–current graphics of (a) ASE3, (b) ASE5-T, and (c) capacitance-scan rate of samples.

**Table 5:** Comparison of Specific Capacitance Values of Biomass-Derived Supercapacitor Materials

biomass source	activation method	electrolyte	specific fapacitance (F/g)	ref
**Apricot Kernel (ASE3)**	KOH + CO_2_	3 M KOH	**136**	this study
**Apricot Kernel** (ASE5-T)	KOH + CO_2_	3 M KOH	**75.5**	this study
coconut shell	KOH	6 M KOH	175.6	^47^
bamboo stalk	KOH	1 M H_2_SO_4_	222	^48^
garlic skin	KOH	1 M H_2_SO_4_	315	^49^
leftover rice	KOH	6 M KOH	153.2	^50^
apricot shell	H_3_PO_4_	6 M KOH	169.1	^51^
sugar beet pulp	KOH	1 M Na_2_SO_4_	142	^52^
tea leaves (waste)	KOH	1 M Na_2_SO_4_	182	^53^

Another important parameter of electrochemical behavior for the capacitor is the voltage–time graphs obtained against the applied current density. Voltage (V)–time (s) graphs of ASE3 and ASE5-T samples are given in Figure 4a,b. Analysis of Figure 4a,b revealed that the ASE3 sample exhibited the longest time interval (∼770 s), whereas the ASES-T sample displayed a shorter duration (∼440 s). Figure 4c shows the specific capacitance behavior of the ASE3 and ASE5-T capacitors as a function of current. While both electrodes reached peak specific capacitance at 1.5 mA/g, the ASE3 electrode demonstrated significantly higher performance. At a scan rate of 5 mV/s, the ASE3 electrode achieved a specific capacitance of 136 F/g, nearly double that of the ASE5-T electrode (75.5 F/g). The long-term cycling stability and Coulombic efficiency (CE) are crucial metrics for evaluating the practical applicability of energy storage devices. As depicted in Figure 4d, both ASE5-T and ASE3 electrodes demonstrate remarkably high Coulombic efficiencies after 5000 cycles, indicating highly effective and reversible charge/discharge processes. Specifically, ASE5-T exhibits an exceptionally high Coulombic efficiency of 99.6% after 5000 cycles at 0.4 mA. This indicates that nearly all of the charge stored is released during discharge, highlighting highly efficient energy conversion. Similarly, ASE3 also maintains an excellent Coulombic efficiency of 98.3% after 5000 cycles. These very high CE values for both materials underscore their significant potential for energy storage applications requiring prolonged cycling and high efficiency.

**Figure 4:**
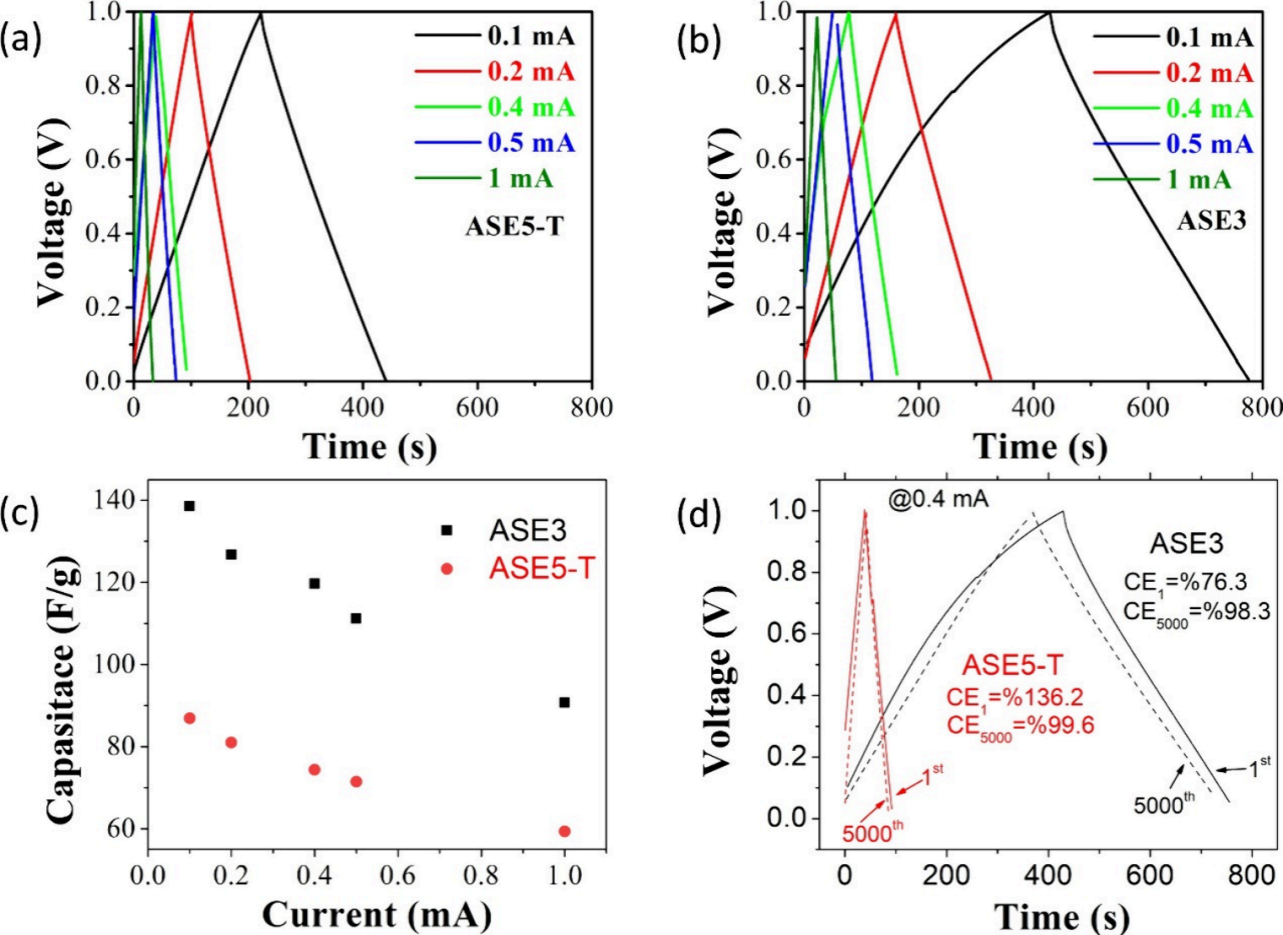
Electrochemical performance of ASE5-T and ASE3 electrodes. (a) Galvanostatic charge–discharge (GCD) curves of ASE5-T at different current densities. (b) GCD curves of ASE3 at different current densities. (c) Specific capacitance as a function of current density for ASE5-T and ASE3. (d) Comparison of GCD curves for the first and 5000th cycles at 0.4 mA for ASE5-T and ASE3, highlighting their capacitance retention (CE).

Figure 5 shows the galvanostatic charge–discharge measurements of ASE3 and ASE5-T samples up to 5000 cycles at a current density of 0.4 mA, along with their corresponding specific capacity values. The capacity retention of ASE3 and ASE5-T is calculated as 96.8% and 93.9%, respectively. The capacitor prepared with the ASE3 sample exhibited the lowest capacity fade, consistent with the results of other electrochemical analyses.

**Figure 5:**
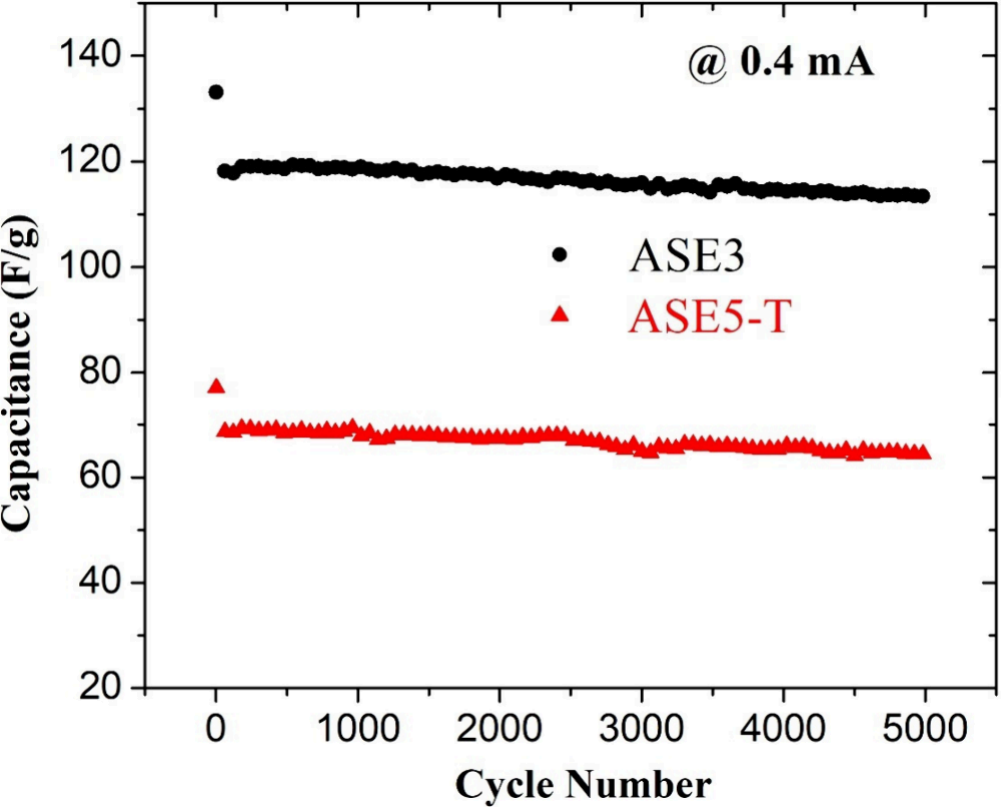
Cycling performance of ASE3 (black circles) and ASE5-T (red triangles) electrodes measured at a current density of 0.4 mA over 5000 cycles.

The impact of residual inorganic impurities on the electrochemical performance of our samples was meticulously examined. As shown in Table 4, the ash content for ASE3 and ASE5-T was measured at 1.36% and 11.72%, respectively. These low percentages suggest that the level of residual inorganic impurities in our synthesized activated carbons is minimal (Table 3). Although these trace impurities, such as silicon and iron, detected by EDX analysis (Figure 2), have the potential to influence electrochemical properties, their low concentration suggests a negligible impact. For instance, the specific capacitance of ASE3 was found to be 136 F/g, while ASE5-T exhibited a specific capacitance of 75.5 F/g (Figure 3(c)). The minor variations in electrochemical performance between the samples can largely be attributed to differences in their specific surface areas and pore size distributions rather than the trace amounts of inorganic impurities.^54^

Although ASE3 shows a remarkably high surface area (2767 m^2^/g), its specific capacitance (136 F/g) remains moderate compared to other biomass-derived carbons reported in the literature (Table 5). This can be attributed to three main factors. First, the high *I*_D_//*I*_G_ ratio (2.55) indicates a disordered carbon structure, which may lower electrical conductivity and hinder charge transport. Second, ASE3 is rich in mesopores, which, despite facilitating ion diffusion, are less efficient in forming electric double layers than micropores. Lastly, some internal pores may be inaccessible due to blockage or tortuosity caused during activation. These factors together may reduce the effective utilization of the available surface area, leading to lower capacitance.

Although the specific capacitance of ASE3 (136 F/g) is lower than some apricot shell–based reports,^55^ the material demonstrates favorable rate capability and long-term stability. These advantages can be attributed to the kernel-derived carbon framework, which provides a compact and conductive structure along with hierarchical porosity. The balanced micro/mesopore distribution facilitates ion transport while maintaining robust structural integrity during cycling.

Electrochemical impedance spectroscopy (EIS) was used to evaluate the electrochemical performance of the ASE3 and ASE5-T electrodes. The data were analyzed using an equivalent circuit model comprising a series resistance (Rs), a charge transfer resistance (R1), two constant phase elements (Q1 and Q2), and a Warburg impedance (W).^56^ Table 6 shows parameters extracted from the fitting process, while the Nyquist plot in Figure 6 visually compares the impedance behavior of the two samples.

**Table 6:** EIS Fitting Parameters of ASE3 and ASE5-T Samples

sample	W (10^–1^)	Rs (10^–1^)	R1 (10^–2^)	Qy1 (10^–5^)	Qy2 (10^–1^)	Qa1 (10^–1^)	Qa2 (10^–2^)
ASE3	5.04	4.62	1.00	8.90	8.19	9.09	4.37
ASE5-T	5.20	6.79	2.00	9.66	8.85	8.59	3.18

**Figure 6:**
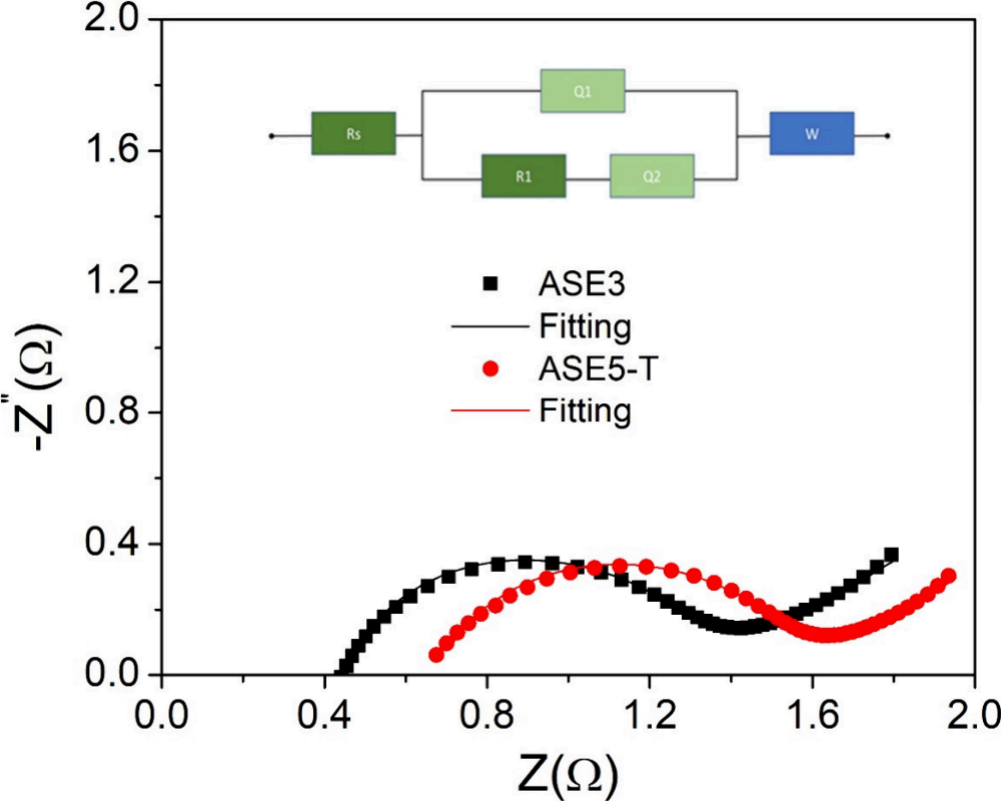
EIS graph of ASE3 and ASE5-T supercapacitor devices. The equivalent circuit used in the fit is shown at the top of the graph.

The series resistance (Rs), which accounts for the combined ohmic resistance of the electrolyte, electrode, and contact points, was found to be lower for ASE3 (4.62 × 10^–1^) than for ASE5-T (6.79 × 10^–1^). This lower resistance indicates that ASE3 exhibits superior conductivity, attributed to better interfacial contact and lower intrinsic resistance within the electrode material. The charge transfer resistance (R1), associated with faradaic processes at the electrode/electrolyte interface, was also significantly lower for ASE3 (1.00 × 10^–2^) compared to ASE5-T (2.00 × 10^–2^). This suggests that ASE3 facilitates faster charge transfer kinetics, enhancing its efficiency during rapid charge/discharge processes. The Nyquist plot supports these findings, as ASE3 shows a smaller semicircle diameter in the high- to high-to-intermediate-frequency region, which directly corresponds to a lower R1 value.^57^ The constant phase elements (Q1 and Q2) further reveal the capacitive behavior of the two electrodes at different frequency ranges. The Q1 values, representing the low-frequency capacitive behavior associated with the double-layer capacitance, were slightly higher for ASE5-T (9.66 × 10^–5^) than ASE3 (8.90 × 10^–5^). Similarly, the intermediate-frequency capacitance described by Q2 was more prominent in ASE5-T (8.85 × 10^–1^) than in ASE3 (8.19 × 10^–1^). These results indicate that ASE5-T demonstrates better charge storage performance at low and intermediate frequencies, which is advantageous for applications requiring extended charge retention and long discharge cycles.

The W, which reflects the ion diffusion resistance within the porous electrode material, showed comparable values for both electrodes, with ASE3 exhibiting a slightly lower value (5.04 × 10^–1^) than ASE5-T (5.20 × 10^–1^). On the other hand, ASE3 exhibits an average pore diameter of 2.0391 nm, close to the size range of solvated ions in aqueous electrolytes (e.g., KOH). This matching pore size enhances ion accessibility and minimizes diffusion resistance, enabling efficient charge storage even at higher scan rates. Conversely, ASE5-T has a slightly larger average pore diameter of 2.1982 nm, which might reduce specific capacitance due to less effective utilization of pore volume for charge storage. Studies have shown that carbon materials with pore sizes narrowly tailored to the size of electrolyte ions achieve higher capacitances by optimizing ion packing within the pores.^58^ This implies better ion transport behavior in ASE3, likely resulting from a more efficient pore structure or improved accessibility of active sites for ion diffusion. In the low-frequency region of the Nyquist plot, the Warburg element is reflected in the linear tail, which confirms the finite diffusion of ions in both systems.^57^

## 3 Conclusions

This study demonstrates the feasibility of utilizing oil-extracted apricot kernel waste, a novel and underutilized biomass, as a sustainable precursor for activated carbon synthesis, with promising performance as supercapacitor electrodes. Elemental analysis confirmed an increase in carbon content at higher carbonization temperatures, while structural characterization revealed the development of porous networks consisting of micropores and mesopores. The ASE3 sample exhibited a high specific surface area (2767.45 m^2^/g) and superior specific capacitance (136 F/g at 5 mV/s), highlighting its potential for efficient ion transport and energy storage applications.

ASE3 exhibits lower series resistance, lower charge transfer resistance, and enhanced ion diffusion, making it more suitable for high-power applications where rapid charge/discharge cycles are required. In contrast, ASE5-T shows enhanced low- and intermediate-frequency capacitive behavior, indicating superior charge storage capability over extended time scales. These differences suggest that ASE3 is better suited for high-power density applications, while ASE5-T is more suitable for energy storage systems that prioritize long-term energy retention.^59^

The variations in electrochemical performance between the ASE3 and ASE5-T samples are attributed to significant differences in their surface areas and elemental compositions (Table 2, Table 3, Figure 2). The ASE3 sample exhibits a substantially higher surface area (2767.45 m^2^/g) and a richer carbon content (78.29%), facilitating greater interaction with electrolyte ions and enhanced charge storage capacity. The ASE5-T sample, in contrast, has a surface area of 1096 m^2^/g, indicating that ASE3 has a 152.5% larger surface area than ASE5-T. Additionally, the charge transfer resistance of ASE3 is 0.01 ohm, which is 50% lower than that of ASE5-T, which is 0.02 ohm. The optimized pore structure enables faster charge/discharge rates and ion diffusion. These findings demonstrate that ASE3 is a more suitable candidate for energy storage applications and that surface area and elemental composition significantly influence electrochemical performance.

These results underscore the potential of biomass-derived activated carbons as low-cost, environmentally friendly materials for advanced energy storage devices. The observed performance, coupled with the abundance and renewability of the raw material, positions these materials as viable alternatives to conventional active materials in supercapacitors.

Although the carbonization temperature of 500 °C yielded favorable porosity and electrochemical performance in this study, future research could explore the effects of higher carbonization temperatures (e.g., 600–700 °C) on the structural evolution and capacitance behavior of apricot-kernel-derived activated carbons. While elevated temperatures may promote greater graphitic ordering and potentially enhance conductivity, they may also lead to pore collapse or reduction in micropore volume, ultimately lowering the surface area and electrochemical efficiency. A systematic investigation into the trade-offs between graphitization, porosity retention, and energy cost at higher carbonization temperatures would provide valuable insights into further optimizing these materials for advanced energy storage applications.

Future research should focus on scaling up the activation process for industrial applications, optimizing the synthesis conditions to further enhance performance, and investigating alternative biomass sources with comparable or superior properties. Furthermore, incorporating these materials into hybrid systems may elucidate novel opportunities for broader energy storage applications. This research contributes to reducing global carbon emissions and addresses critical energy challenges by advancing the development of environmentally sustainable energy storage technologies.

## 4 Methods

### Material Preparation

The primary raw material for this study was oil-extracted apricot kernel waste sourced from the Malatya region. The kernels were defatted using a cold press machine, and the residual apricot seed (ASE) pulp was used directly in the experiments without further processing. ASE3 refers to the sample carbonized at 500 °C, while ASE5-T represents the sample carbonized at 400 °C and activated under identical conditions.

### Carbonization Process

The defatted seed pulp was carbonized in a three-zone cylindrical furnace to prepare the ASE3 and ASE5-T samples. The samples were placed in steel reactors under a nitrogen atmosphere at a pressure of 1 atm to prevent undesirable reactions. The furnace temperature was gradually increased at a rate of 10 °C/min, reaching 400 °C for ASE5-T and 500 °C for ASE3. Each sample was held at its respective target temperature for 24 h. After cooling, the carbonized solids were retrieved for subsequent activation.

### Activation Process

The carbonized samples were impregnated with KOH in a 1:3 weight ratio and placed in a glass reactor for chemical activation. Activation was conducted in a three-zone furnace at 800 °C under a controlled CO_2_ flow (100 mL/min). Here, CO_2_ was not used solely to maintain an inert atmosphere but rather to serve as a physical activating agent. The CO_2_ gas promotes additional porosity development through gasification reactions with carbon (C + CO_2_ → 2CO), thereby enhancing the effects of chemical activation and contributing to a hierarchical pore structure.^60^ This combined activation approach is known to improve both the specific surface area and the pore accessibility of the resulting carbon materials.

The heating rate was maintained at 10 °C/min, and the final temperature was held for 1 h. Following activation, the furnace was cooled under argon flow (100 mL/min) to room temperature over 24 h. The mixture removed from the oven was first treated with 0.1 M HCl solution and boiled to neutralize the residual KOH. Subsequently, the samples were thoroughly washed with deionized (DI) water until the pH of the filtrate reached ∼7. The absence of residual chloride was confirmed using a silver nitrate (AgNO_3_) test. Finally, the washed samples were dried at 100 °C and stored in sealed plastic containers for further analyses.

### Ash Content Analysis

The synthesized activated carbon samples were analyzed for ash content. The samples were placed in porcelain crucibles and heated in a staged process, initially at 650 °C and subsequently at 850 °C until complete carbon combustion was achieved. The residual ash content was determined and is reported in Table 4.

### Structural Characterization

X-ray diffraction (XRD) measurements were performed on the synthesized samples to determine their structural characteristics, specifically to identify whether they exhibited crystalline or amorphous properties. XRD analysis of the samples was performed using Cu–Kα radiation on a Rigaku RadB Dmax diffractometer. PerkinElmer Spectrum One Model (400–4000 cm^–1^) Fourier transform infrared spectroscopy (FTIR) analyses were performed to determine the bond structures of the samples. Raman spectra of the samples were collected using a 532 nm laser under the Renishaw in Via Qontor, Malatya, Turkey. Scanning electron microscopy (SEM) coupled with energy-dispersive X-ray spectroscopy (EDX) was employed to examine the morphology of the samples and their chemical composition. To calculate the surface area of the samples, the Brunauer-Emmet-Teller (BET) technique was performed with the help of Micromeritics-TriStar 3000.

### Electrochemical Characterization

The supercapacitor electrodes were composed of activated carbon as the active material, carbon black as the conductive additive, and polyvinylidene fluoride functioning as the binder (80:10:10 w/w/w). *N*-Methyl-2-pyrrolidone (NMP) was utilized as the solvent for the binder and mixed in a magnetic stirrer for 24 h until a fluid and homogeneous mixture was obtained. Once the mixing process was complete, the mixture was transferred to an ultrasonic bath and left for 10 min to ensure complete homogenization before use. The electrode mixture was applied to the Ni foam current collector using a laboratory tape-casting machine. The coated Ni foam was then dried at 75 °C for 24 h under vacuum to remove any residual solvent. The electrode films and the commercial Celgard polypropylene separator were cut into circular shapes with 15 mm and 18 mm diameters, respectively. Subsequently, the disc-shaped electrodes and separators were immersed in electrolyte solutions, specifically 3 M KOH/aqueous. The CR2032 coin-type supercapacitor devices were assembled and sealed by positioning the electrolyte-soaked separator between two identical electrodes to construct a sandwich-type supercapacitor.

Cyclic voltammetry (CV) analyses were performed between 0 and 1.0 V voltage ranges using different scanning rates (5–200 mV/s). Charge/discharge measurements were conducted at room temperature and within the range of 0.0–1.0 V, using the constant current (CC) method at 0.4 mA current density. In addition to all these analyses, capacitance values of current density were also determined. EIS analysis of the samples was performed under open-circuit voltage with a logarithmic scanning rate from 100 mHz to 200 kHz. All electrochemical characterization measurements were performed with a Zive SP1 device.

## Data Availability

The data underlying this study are available in the published article.
